# Genetics Underlying Atypical Parkinsonism and Related Neurodegenerative Disorders

**DOI:** 10.3390/ijms161024629

**Published:** 2015-10-16

**Authors:** Sonja W. Scholz, Jose Bras

**Affiliations:** 1 Neurodegenerative Diseases Research Unit, Laboratory of Neurogenetics, National Institute of Neurological Disorders and Stroke, National Institutes of Health, 35 Convent Drive, Bethesda, MD 20892, USA; 2 Department of Neurology, Johns Hopkins University School of Medicine, 1800 Orleans Street, Baltimore, MD 21287, USA; 3 Department of Molecular Neuroscience, University College London, Institute of Neurology, Queen Square House, London WC1N 3BG, UK; E-Mail: j.bras@ucl.ac.uk

**Keywords:** atypical parkinsonism, multiple system atrophy, dementia with Lewy bodies, progressive supranuclear palsy, corticobasal degeneration

## Abstract

Atypical parkinsonism syndromes, such as dementia with Lewy bodies, multiple system atrophy, progressive supranuclear palsy and corticobasal degeneration, are neurodegenerative diseases with complex clinical and pathological features. Heterogeneity in clinical presentations, possible secondary determinants as well as mimic syndromes pose a major challenge to accurately diagnose patients suffering from these devastating conditions. Over the last two decades, significant advancements in genomic technologies have provided us with increasing insights into the molecular pathogenesis of atypical parkinsonism and their intriguing relationships to related neurodegenerative diseases, fueling new hopes to incorporate molecular knowledge into our diagnostic, prognostic and therapeutic approaches towards managing these conditions. In this review article, we summarize the current understanding of genetic mechanisms implicated in atypical parkinsonism syndromes. We further highlight mimic syndromes relevant to differential considerations and possible future directions.

## 1. Introduction

The term atypical parkinsonism refers to a heterogeneous group of neurological disorders characterized by the presence of parkinsonism plus additional “atypical” features for Parkinson disease (PD), such as early cognitive impairment, autonomic dysfunction, early falls, ataxia, apraxia, dystonia, myoclonus, amyotrophy, gaze palsy, cortical sensory loss, alien limb phenomenon or failure to respond to dopaminergic treatment. This group of conditions used to be referred to as Parkinson-Plus syndromes due to the presence of additional characteristics, though this term has fallen out of favor. The accurate clinical diagnosis of atypical parkinsonism syndromes is challenging due to phenotypic variability, overlapping clinical features between neurodegenerative disorders and a lack of disease-specific diagnostic tests. The net result of this is that the correct diagnosis is often only established at autopsy.

The last two decades have seen tremendous progress in our understanding of the genetic etiology of neurological disease. In large part, this is due to advances in genomic technologies that have allowed us to systematically unravel the genetic architecture of disease. In neurodegenerative diseases research, increasing genetic evidence demonstrates overlapping risk loci between atypical parkinsonism syndromes, suggesting that these syndromes are pathogenetically related. Ultimately, this knowledge has and continues to identify new targets for therapeutic development, and the long-term hope is that it will ultimately lead to effective treatments for patients devastated with these disorders.

In this review article, we provide an overview of the current state of genetic knowledge in atypical parkinsonism. We focus on the sporadic forms of atypical parkinsonism, such dementia with Lewy bodies (DLB), progressive supranuclear palsy (PSP), corticobasal degeneration (CBD), and multiple system atrophy (MSA). Unusual presentations of common neurodegenerative diseases that may mimic atypical parkinsonism syndromes will also be discussed. Finally, we will highlight future directions and implications for therapeutic interventions.

## 2. Multiple System Atrophy

Multiple system atrophy (MSA) is an adult-onset, progressive neurodegenerative disorder that presents with variable combinations of parkinsonism, cerebellar ataxia, autonomic failure and pyramidal signs. MSA affects men and women equally with a mean age at onset of 56 years and a median survival of 10 years [1,2]. The incidence of MSA is about 3 per 100,000 per year (age range: 50 years or older) and the prevalence is 4.4 per 100,000 [3,4]. Clinical presentation is influenced by ethnic background, with parkinsonism more commonly seen in Caucasians and cerebellar features more frequently observed in the Japanese population [5,6].

Histopathologically, MSA patients have characteristic oligodendroglial cytoplasmic inclusions (GCIs) made up of misfolded, hyperphosphorylated α-synuclein filaments [7,8]. This neuropathological hallmark classifies MSA under the rubric of synucleinopathies, a diverse group of neurodegenerative diseases characterized by abnormal deposition of misfolded α-synuclein [9]. GCI formation, neuronal loss, reactive astrocytic gliosis and microglia activation mainly affect the central autonomic, olivopontocerebellar and striatonigral structures, explaining the variable clinical presentations of patients suffering from this fatal condition [10]. There is increasing evidence suggesting that α-synuclein deposition occurs in a step-wide fashion [11]. Myelin-to-cytoplasm relocalization of p25α, a microtubule stabilizing protein, is one of the first events [12]; oligodendroglial swelling, α-synuclein oligomerization and fibrillogenesis follow this initial process [13]. Biochemical analyses suggest that some misfolded synuclein species are toxic and propagate to associated neurological pathways using a prion-like mechanism, which results in characteristic multi-system involvement [14]. A recent, controversial study even concluded that MSA is a prion disease [15]. This study was based on the observation that cells and mouse models carrying a pathogenic synuclein mutation can form synuclein aggregates when treated with brain homogenate from MSA patients. The main critique regarding this study is that no transmission from an affected individual to a healthy recipient was demonstrated, and such transmissibility is generally considered a defining feature of prion diseases. Furthermore, there are also no epidemiologic data suggesting that MSA is a prion disease. It is curious though that cells carrying pathogenic synuclein mutations were prone to develop aggregates, which could indicate that a genetic predisposition could play a role in the etiopathogenesis, perhaps by establishing a cellular milieu that facilitates aggregation. The exact process by which aggregated synuclein subsequently leads to neurodegeneration, however, is incompletely understood.

Rare pathology-proven MSA families following either autosomal recessive or autosomal dominant patterns have been described, indicating a genetic contribution to at least a subset of patients [16,17,18,19,20]. Interestingly, parkinsonism is more common in relatives of MSA patients. This observation also supports the hypothesis of a genetic predisposition to the pathogenesis of MSA [21,22].

Recessive mutations in *COQ2* were recently reported in Japanese multiplex MSA families and heterozygosity for the common V393A polymorphism in the same gene was nominated as a susceptibility variant for sporadic cases [17]. Nevertheless, the role of *COQ2* variants in the pathogenesis of MSA is a topic of debate in the field as independent replications in Caucasians and South Korean cohorts failed to demonstrate association with disease [23,24,25,26]. Furthermore, loss-of-function mutations in *COQ2* had been previously shown to cause primary co-enzyme Q10 deficiency, a severe multisystem infantile syndrome characterized by variable presentations of encephalopathy, epilepsy, psychomotor regression, retinopathy, myopathy and nephropathy [27]. It remains possible that the described variants in the original families represent spurious associations.

The neuropathological characteristics in MSA have led to an intense search for mutations in the *SNCA* gene, which codes for α-synuclein. Further impetus to this search came from the observation that missense mutations, triplications and duplications in *SNCA* are a known, rare cause of autosomal dominant PD that occasionally presents with clinical and pathological features similar to MSA [28,29,30]. Candidate gene sequencing, expression and copy number studies in autopsy-confirmed MSA cases, however, have not demonstrated any mutations, expression level or copy number changes in *SNCA* [31,32,33,34,35,36].

Following a genome-wide association study in PD demonstrating significant association at the *SNCA* locus, we examined the most significant variants of this study in a large MSA cohort and matched controls [37,38]. Significant associations of *SNCA* variants with risk for developing MSA were found and these results were subsequently replicated in Caucasian cohorts [39,40]. Similar associations were not detected in Asian populations [41,42,43]. These data support the notion that common variability at the *SNCA* locus is a risk factor for sporadic MSA in Caucasians, but population-dependent heterogeneity likely explains that lack of association in Asian populations. From a practical perspective, consideration for *SNCA* screening in patients with a clinical diagnosis of MSA should only be given if a strong, autosomal dominant family history of PD, DLB or MSA is present.

Numerous additional candidate genes, most notably *MAPT*, *PRNP*, genes linked to spinocerebellar ataxias (SCA), genes linked to PD or DLB, and genes involved in neuroinflammation have been explored, with mixed results [44]. The *APOE* ε*4* allele, which is a known significant risk factor for Alzheimer dementia (AD) and for DLB, is not associated with MSA [45]. Positive results indicating association with disease were reported for the following genes: G*BA*, *LRRK2*, *MAPT*, *SCA3*, *SLC1A4*, *SQST1*, *EIF4EBP*, *IL-1a*, *IL-1b*, *TNF*, *IL-8*, *ICAM1*, *ACT*, *ADH1C*, *SHC2* [46,47,48,49,50,51,52,53,54,55,56,57]. However, many of the studies were based on small sample sizes, yielded conflicting results in replication attempts or lack independent replications, raising the concern of false positive results. These reports should therefore be interpreted with caution until additional replication studies are performed.

A major challenge in the search for genes underlying MSA is the difficulty in establishing an accurate clinical diagnosis. A wide range of diseases can clinically mimic the MSA phenotype (Table 1) [28,30,58,59,60,61,62,63,64,65,66,67,68,69,70,71,72,73,74,75,76,77,78,79,80,81,82,83,84,85,86,87,88,89,90,91,92,93,94,95,96,97,98,99,100,101,102], and neuropathological series have shown that the accuracy of a clinical MSA diagnosis can vary widely (from 25% to 86%) [103,104,105]. Thus, there is still a critical need for identifying biomarkers, such as genetics, molecular signatures or distinct neuroimaging modalities, which will improve the clinical diagnostic accuracy. This is particularly true for the cerebellar MSA variant that can be indistinguishable from spinocerebellar ataxia syndromes, especially in an early disease stage [61,62,64,65,66,106]. Further, patients with coding or copy number mutations in *SNCA* or patients carrying a *GBA* mutation can occasionally present with an MSA-like phenotype [28,30,72,76,77]. Patients with pathologic hexanucleotide repeat expansions in *C9orf72*, a gene linked to amyotrophic lateral sclerosis (ALS) and frontotemporal dementia (FTD), can rarely demonstrate clinical and neuroimaging features indistinguishable from MSA [58]. Ideally, a detailed family history should point the clinician towards a hereditary condition. However, lack of family history cannot be taken as definite proof against a genetic predisposition/causation, given various potential genetic events, such as reduced penetrance, the occurrence of spontaneous mutations, phenotype variability, death of relatives prior to symptom onset or non-paternity.

**Figure 1:**
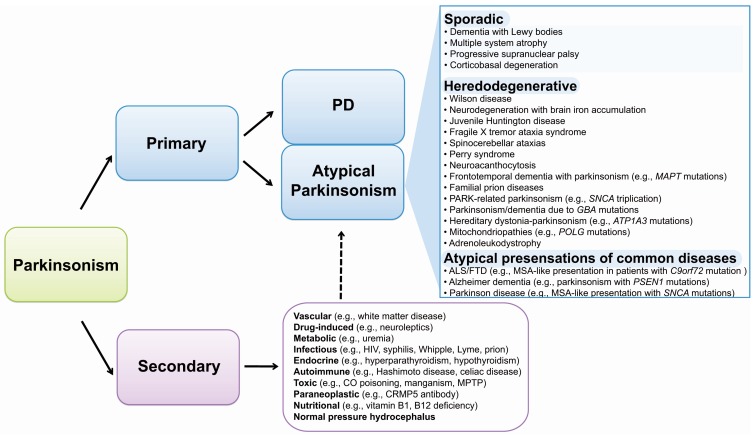
Schematic illustration of parkinsonism syndromes. Gene names are shown in italic font. Solid arrows indicate major classification groups; dashed arrow points out a possible interplay between primary and secondary parkinsonism. Abbreviations: PD, Parkinson disease; ALS/FTD, amytrophic lateral sclerosis/frontotemporal dementia; HIV, human immunodefiency virus; CO, carbon monoxide; MPTP, 1-methy-4-phenyl-1,2,3,6-tetrahydropyridine.

Aside from hereditary disorders that can present with MSA-like features, consideration should also be given to secondary, potentially treatable causes of atypical parkinsonism, such as exposure to neuroleptic medications, cerebrovascular insults, endocrine dysfunction (e.g., hypothyroidism, hyperparathyroidism), infectious diseases (e.g., Lyme disease), autoimmune conditions (e.g., Hashimoto disease, celiac disease), toxic syndromes (e.g., manganism, superficial siderosis), neoplastic processes (e.g., gliomatosis cerebri) or paraneoplastic syndromes (e.g., anti-Ma2 antibody) (Figure 1) [107,108,109,110,111,112,113].

**Table 1:** Heredodegenerative disorders that can clinically mimic sporadic atypical parkinsonism syndromes.

Typical Presentation	Gene	Chr.	Inheritance	Mutation Type	Mimic Syndrome(s)	Ref.
ALS-FTD	*C9orf72*	9p21.2	AD	Repeat Expansion	MSA, PSP, CBS	[58,59]
FA	*FXN*	9q21.11	AR	Repeat Expansion	MSA	[60]
SCA 2	*ATXN2*	12q24.1	AD	Repeat Expansion	MSA	[62]
SCA 3	*ATXN3*	14q21	AD	Repeat Expansion	MSA	[61]
SCA 6	*CACNA1A*	19p13	AD	Repeat Expansion	MSA	[60]
SCA 7	*ATXN7*	3p21.1-p12	AD	Repeat Expansion	MSA	[106]
SCA 8	*ATXN8*	13q21	AD	Repeat Expansion	MSA, CBS	[64]
SCA 17	*TBP*	6q27	AD	Repeat Expansion	MSA, PSP	[65]
SCA 23	*PDYN*	20p13	AD	Sequence Mutation	MSA	[66]
FXTAS	*FMR1*	Xq27.3	XR	Repeat Expansion	MSA	[67]
ALD	*ABCD1*	Xq28	XR	Sequence Mutation, Deletion	MSA	[102]
PD	*SNCA*	4q21	AD	Sequence Mutation, Triplication/Duplication	MSA, DLB	[28,30,68,69]
PD	*LRRK2*	12q12	AD	Sequence Mutation	PSP, CBS	[73,74]
Gaucher disease/PD	*GBA*	1q21	AR/AD	Sequence Mutation, Deletion, Insertion	PSP, CBS, LBD	[76,77]
Perry syndrome	*DCTN1*	2p13	AD	Sequence Mutation	MSA, CBS, PSP	[78,79]
CTX	*CYP27A1*	2q35	AR	Sequence Mutation, Deletion, Duplication	MSA, PSP, CBS	[80,81]
Mitochondriopathy	*POLG1*	15q25	AR/AD	Sequence Mutation	MSA	[82]
AD	*PSEN1*	14q24.3	AD	Sequence Mutation, Deletion	DLB, CBS	[83,84]
AD	*PSEN2*	1q42.13	AD	Sequence Mutation	DLB	[85]
AD	*APP*	21q21.3	AD	Sequence Mutation, Duplication	DLB	[86]
KRS	*ATP13A2*	1p36	AR	Sequence Mutation	PSP	[87]
FTD	*GRN*	17q21.32	AD	Sequence Mutation	PSP, CBS	[88,89,90]
FTD	*TARDBP*	1p36.22	AD	Sequence Mutation	PSP, CBS	[91]
FTD	*FUS*	16p11.2	AD	Sequence Mutation	PSP, CBS	[92]
CADASIL	*NOTCH3*	19p13.2-p13.1	AD	Sequence Mutation	PSP	[94]
NPC	*NPC1*; *NPC2*	18q11.2; 14q24.3	AR	Sequence Mutation	PSP	[95,96]
FTD	*MAPT*	17q21.1	AD	Sequence Mutation	PSP, CBS	[97,98]
Familial prion disease	*PRNP*	20p13	AD	Sequence Mutation	PSP, CBS	[99,100]
HDLS	*CSF1R*	5q32	AD	Sequence Mutation	CBS	[101]

Abbreviations: ABCD1, encoding ATP-binding cassette subfamily D member 1; AD, Alzheimer dementia or autosomal dominant; ALD, adrenoleukodystrophy; ALS-FTD, amyotrophic lateral sclerosis/frontotemporal dementia; APP, encoding amyloid beta precursor protein; AR, autosomal recessive; ATP13A2, encoding ATPase type 13A2; ATXN2, encoding ataxin 2; ATXN3, encoding ataxin 3; ATXN7, encoding ataxin 7; ATXN8, encoding ataxin 8; CACNA1A, encoding voltage-dependent calcium channel alpha 1A subunit; CADASIL, cerebral autosomal dominant arteriopathy with subcortical infarcts and leukencephalopathy; CBS, corticobasal syndrome; Chr., chromosome; CSF1R, encoding colony stimulation factor 1 receptor; CTX, cerebrotendinous xanthomatosis; CYP27A1, encoding cytochrome P450 family 27 subfamily A polypeptide 1; C9orf72, encoding chromosome 9 open reading frame 72; DCTN1, encoding dynactin 1; FA, Friedreich ataxia; FMR1, encoding fragile X mental retardation 1; FTD, frontotemporal dementia; FUS, encoding FUS RNA binding protein; FXN, encoding frataxin; FXTAS, fragile X tremor ataxia syndrome; GBA, encoding glucocerebrosidase β; GRN, encoding granulin; HDLS, hereditary diffuse leukencephalopathy with spheroids; KRS, Kufor Rakeb syndrome; DLB, dementia with Lewy bodies; LRRK2, encoding leucine-rich repeat kinase 2; MAPT, encoding microtubule-associated protein tau; MSA, multiple system atrophy; NOTCH3, encoding notch 3; NPC, Niemann-Pick type C; NPC1, encoding Niemann-Pick type C1; NPC2, encoding Niemann-Pick type C2; PD, Parkinson disease; PDYN, encoding prodynorphin; POLG1, encoding polymerase γ; PRNP, encoding prion protein; PSEN1, encoding presenilin 1; PSEN2, encoding presenilin 2; PSP, progressive supranuclear palsy; Ref., references; SCA, spinocerebellar ataxia; SNCA, encoding α-synuclein; TARDBP, encoding TAR DNA binding protein; TBP, encoding TATA box binding protein; XR, X-linked recessive.

## 3. Dementia with Lewy Bodies

DLB is the second most common neurodegenerative dementia in the elderly population, accounting for about 20% of all dementias in autopsy series [114]. The condition is characterized by variable combinations of progressive cognitive decline, parkinsonism, fluctuating mental status, neuroleptic sensitivity and visual hallucinations [115]. The term Lewy body dementia is used as an umbrella term to embrace two closely related clinical entities, namely dementia with Lewy bodies (DLB) and Parkinson disease dementia (PDD). According to international consensus criteria, the diagnosis of PDD is established if motor symptoms manifest more than a year prior to onset of cognitive dysfunction. In the case of cognitive features preceding motor symptoms or cognitive symptoms occurring within one year of motor problems, the diagnosis of DLB is given [116]. There are ongoing debates whether splitting Lewy body dementia into DLB and PDD is practical, particularly in light of similar pathological findings in these two patient populations, as well as recall bias and referral bias influencing the choice of terms used.

As in PD, the histopathology in DLB patients shows Lewy bodies and Lewy neurites comprised of aggregated α-synuclein. In contrast to PD though, α-synuclein in DLB patients is more widespread with neocortical, limbic and brainstem involvement [117]. Pathological features of AD in the form of neurofibrillary tangles and amyloid plaques are observed in the majority of patients, placing DLB along a spectrum between PD and AD. Neuronal and synaptic loss is pronounced in the substantia nigra, amygdala, locus coeruleus, nucleus basalis Meynert, dorsal nucleus of vagus and nucleus ambiguus, resulting is multiple neurotransmitter abnormalities involved in motor processing, cognition and autonomic nervous system control [117,118].

Although DLB is generally considered a sporadic disease of late adulthood, rare familial occurrences do suggest a genetic predisposition in at least a subset of cases [119,120,121,122,123]. Further evidence in support of a heritable component is derived from observations in PD, which can rarely present in a familial, autosomal dominant fashion due to mutations in *SNCA* (duplication, multiplication or missense mutations) [28,30,68,69,70,71,72,124]. Affected individuals occasionally present with clinicopathological features most consistent with DLB, although significant phenotypic and pathological heterogeneity within these families exists. Another important finding is the observation that mutations in *GBA*, encoding for the lysosomal enzyme glucocerebrosidase, significantly increase risk for PDD and for DLB [125]. This discovery was again driven by observations coming from the PD field, where *GBA* mutations have been firmly established as a risk gene. Taken together, these intriguing observations in *SNCA* families and *GBA* mutation carriers support the notion of shared pathogenic mechanisms leading to these diverse synucleinopathies.

It is important to highlight that the *APOE ε4* allele is a significant risk factor for DLB [126,127]. *APOE* is the strongest known risk gene for sporadic AD, but interestingly it is not associated with risk for PD in a large multicenter GWAS [128]. Multiple other candidate genes implicated in the pathogenesis of neurodegeneration have been explored. Mutations in *APP*, a gene linked to rare familial early-onset AD, have been shown to occasionally present with clinical and pathological features consistent with DLB [86,129]. Along the same lines, mutations in the rare familial AD genes *PSEN1* and *PSEN2* can be associated with Lewy body pathology in addition to typical AD changes [83,130,131]. These genetic observations, together with overlapping clinicopathological features firmly place DLB along a spectrum between PD and AD [132].

## 4. Progressive Supranuclear Palsy

Progressive supranuclear palsy is a neurodegenerative syndrome of unknown etiology clinically characterized by variable combinations of gait impairment, early postural instability, axial rigidity, bradykinesia, ataxia, slow vertical saccades progressing to supranuclear vertical ophthalmoplegia, pseudobulbar palsy and frontal executive dysfunction [133,134]. The age-adjusted prevalence of PSP is estimated to be about 6.4 per 100,000, making it the second most common neurodegenerative form of parkinsonism after PD [135]. PSP typically manifests in the seventh decade of life, and it affects men and women equally. Progression is gradual with a median survival time of 5.6 years [136].

Pathologically, PSP patients have abnormal neuronal and glial neurofibrillary tangles and neuropil threads, which are composed of aggregated, hyperphosphorylated microtubule associated protein tau, predominantly in its four-repeat isoform [137]. Tau protein inclusions are a hallmark of a diverse group of neurologic conditions, commonly referred to as tauopathies, including AD, PSP, CBD, a type of frontotemporal dementia, traumatic encephalopathy, post-encephalitis parkinsonism and parkinsonism-dementia complex of Guam. Brain areas that are most affected in PSP are brainstem, diencephalon and basal ganglia [138]. Microscopically, characteristic tufted astrocytes as well as oligodendroglial coiled bodies are found [139]. Swollen, “ballooned” neurons can be found in PSP, predominantly in paralimbic areas, but they are sparse compared to CBD [140]. Atrophy of above mentioned brain regions leads to multiple neurotransmitter abnormalities, involving the dopaminergic, GABAergic, cholinergic, and serotoninergic systems [141,142,143,144]. Mitochondrial dysfunction results in oxidative stress, which promotes the formation of tau filaments, but the exact mechanism leading to neuronal cell death remains unclear [145].

PSP occurs typically as a sporadic condition, but rare autosomal dominant cases have been documented, predominantly due to mutations in *MAPT* [97,146,147,148]. An additional locus for familial, autosomal dominant PSP has been nominated on 1q31.1, but independent replication has not yet been performed to confirm this finding [149]. Genetic analysis in sporadic PSP cases has yielded exciting insights into the pathogenesis. One of the most striking findings is a strong, reproducible disease association of the H1 haplotype surrounding *MAPT*, the gene that codes for microtubule associated protein tau [150,151,152]. This particular haplotype is made up of a large inversion polymorphism, which is common in the general Caucasian population (78% of chromosomes), but it is significantly overrepresented in PSP patients (~95% of chromosomes) [152]. Although the H1 haplotype significantly increases risk for disease, carrier status is not correlated with age at onset, disease severity or survival [153]. *MAPT* transcripts normally undergo alternative splicing, resulting in several isoforms, of which those containing exon 10 give rise to four-repeat tau. In contrast to normal human brain, in which four-repeat and three-repeat tau are expressed at similar levels, the balance is shifted towards the more aggregation-prone four-repeat isoforms in PSP patients [154,155]. Interestingly, the H1 haplotype has been shown to increase transcription and exon 10 splicing [38,156]. It is therefore possible that increased risk for PSP in H1 haplotype carriers is mediated by a shift towards four-repeat tau.

A large, multicenter genome-wide association study (GWAS) in PSP confirmed the role of H1 *MAPT* haplotype as a major risk factor; in addition several new risk loci were identified: *STX6* (coding for syntaxin 6), *EIF2AK3* (encoding PERK) and *MOBP* (coding for myelin-associated oligodendrocyte basic protein) on chromosomes 1, 2 and 3 respectively [152]. Sequencing of these genes in a subset of pathologically confirmed cases from this study found no coding changes. This finding could indicate that disease association is due to epigenetic drivers surrounding this locus. Interestingly, the risk allele in *STX6* was found to be a strong expression quantitative trait locus, lowering expression of syntaxin 6 in the white matter [157]. Syntaxin 6 is a member of the SNARE protein family that is involved in intra-cellular membrane trafficking. It is localized to the trans-Golgi network and endosomal structures, where it is thought to play a role in endosomal membrane fusion events [158]. Impaired intracellular trafficking of misfolded tau protein, among other pathogenic events, may be a mechanism leading to abnormal tau aggregation. This intriguing hypothesis still has to be further investigated.

Candidate gene approaches have implicated a role of *VEGF*, *NAT2* and *Park2* in the pathogenesis of PSP. However, none of these loci were associated with disease in a large GWAS, suggesting that these associations either escaped detection due to small effect size or the initial results were spurious in nature [152,159,160,161]. A risk locus on 11p12-11, containing the genes *DDB2* and *ACP2*, has been suggested in a pooled GWAS including only 288 pathologically confirmed cases and 344 matched controls, but this locus was not detected in a larger GWAS [152,162]. In the absence of robust replications these loci should therefore be interpreted as false positives.

Numerous genes implicated in variable neurodegenerative diseases, such as PD, ALS, FTD and other atypical parkinsonism syndromes, can mimic the PSP phenotype (Table 1) [59,65,73,75,76,77,78,79,80,87,88,89,90,91,92,93,94,95,96,97,98,99,100,101]. With the exception of certain mutations in *MAPT*, which can be a rare cause of familial PSP, candidate screening studies in some of these mimic genes have been disappointing [59,148,163,164,165,166]. Of note, the *APOE* ε*4* allele, which plays a significant role in AD and DLB, is not associated with PSP [167,168,169]. *APOE* ε*4* carrier status also has no influence on age at onset, disease duration or age at death [170,171].

The clinical presentation of PSP can be variable, explaining the difficulties to accurately diagnose PSP patients, particularly those with uncommon features. Atypical features that are well documented include non-fluent primary progressive aphasia, cortical sensory impairment or FTD. Further, sporadic neurodegenerative diseases, such as PD, AD, MSA, Creutzfeldt-Jakob disease (CJD), ALS and CBD, can rarely also present with PSP-like features and result in a false diagnosis [172,173]. Along the same lines, secondary causes mimicking PSP (e.g., vascular dementia, neurosyphilis, Whipple’s disease, paraneoplastic syndrome, post-encephalitic disease) need consideration when clinically evaluating patients with presumed PSP [174,175,176,177,178,179].

## 5. Corticobasal Degeneration

CBD is a tauopathy that shares molecular and clinical features with PSP. CBD is a sporadic disease of late adulthood that typically presents with variable combinations of progressive, asymmetric parkinsonism, dystonia, apraxia, myoclonus, cortical signs and cognitive impairment. It is important to highlight the difference between the terms corticobasal syndrome (CBS), which refers to this classical clinical presentation, and corticobasal degeneration (CBD), a term used by pathologists to describe this tauopathy based on characteristic histopathological features. The caveat with clinically diagnosed patients is that a heterogeneous group of other neurodegenerative diseases can also occasionally cause this presentation. These include AD, PSP, FTD, DLB and rarely CJD or spinocerebellar ataxia [180,181,182,183,184,185]. Moreover, secondary causes for CBS are well described (including vascular dementia, neurosyphilis, Fahr’s disease, progressive multifocal leukencephalopathy and antiphospholipid syndrome) and need thoughtful consideration during clinical workup of a patient [186,187,188,189,190,191]. Heterogeneity in the clinical appearance of the larger CBD group has also been described, with variable symptoms such as progressive nonfluent aphasia, behavioral-variant FTD, posterior cortical atrophy and PSP-like presentations [192]. Not surprisingly, this complex clinical heterogeneity results in a low clinical accuracy to diagnose CBD patients effectively (Table 1) [193]. In fact, autopsy studies have shown that only about half of patients presenting with CBS have CBD [180,192,194].

Epidemiologic data are limited, but they show that the incidence of CBD is less than 1 per 100,000 people per year and the prevalence is 6 per 100,000. These numbers are likely to be overestimates as patients could present with CBS due to other etiologies [195,196,197]. The mean age at disease onset is 64 years with an average survival time of 7 years (range 2–12.5 years) [198]. No clear gender bias has been observed.

Neuropathologically, CBD is characterized by focal, sometimes asymmetric, cortical atrophy that is most marked in parasagittal regions, commonly affecting the dorsal prefrontal and peri-Rolandic regions, striatum and brainstem [140,192]. The distribution can be more generalized in cases with dementia and progressive aphasia, affecting inferior frontal and temporal lobes. Histopathologically, spongiosis, tau-positive astrocytic plaques and extensive thread-like lesions are found in cortical, basal ganglia and subcortical regions, as well as swollen “ballooned” neurons. Coiled bodies, which are tau-positive oligodendroglial lesions more commonly found in PSP, are also noted [140].

Due to shared clinical, genetic and biochemical characteristics between PSP and CBD, it remains an issue of debate whether these two disease entities are part of a spectrum or are genuinely separate conditions. Similar to PSP, patients with CBD are more likely to carry the H1 *MAPT* haplotype [199]. Both conditions are characterized by tau-positive inclusions consisting predominantly of hyperphosphorylated four-repeat isoforms; however, proteolytic processing appears to be different between these two diseases. Specifically, detergent-insoluble cleaved tau from CBD brain tissue migrates at two bands of approximately 37 kDa, as opposed to a single band observed in brain extracts from PSP patients, which migrates as a 33 kDa band [200]. The explanation behind the various species of tau degradation products remains unclear.

Familial occurrences of CBD have been documented [201,202,203,204,205]. In rare instances, coding mutations in *MAPT* have been found in some of these pathologically confirmed cases [205,206,207], but for most of the familial CBD cases the underlying genetic defect has not yet been established. An exome sequencing study in a CBD family with two affected cousins described coding variants in *AGBL5*, *FANCL*, *PLEKHB2*, *MRS2* and *ZHX2* in both patients. In-silico analyses suggested that the variants in *AGBL5*, *FANCL* and *PLEKHB2* are benign, whereas mutations in *MRS2* and *ZHX 2* are predicted to be damaging to the protein function [202]. The significance of this observation is questionable, particularly given the small size of this family and the fact that these mutations are also reported in the Exome Variant Server, indicating that they could just be rare polymorphisms in the population. No independent study has yet investigated these genes.

Data from a recent small-sized GWAS support the notion of a genetic contribution to the pathogenesis of the more common sporadic form of CBD [208]. Not surprisingly, this study confirmed association with *MAPT*. In addition, a significant signal was noted in an intronic SNP within the non-coding RNA *lnc-KIF13B-1* on 8p12. This lnc RNA regulates transcription of KIF13B, a microtubule based motor protein involved in vesicular trafficking along microtubules, axon extension and caveolin-dependent endocytosis [209,210,211]. Additional loci were suggested on 2p22 and 3p22, but these signals did not meet genome-wide significance. It is of interest though that the signal on 3p22 is at the *MOBP* locus, a gene that has been previously linked to PSP [152]*.* This observation supports the notion that PSP and CBD are closely related on a molecular level. *EIF2AK3* and *STX6*, which are two other loci from a previous PSP GWAS, were not found to be associated with CBD. While these findings are promising, independent replication is required to confirm these observations.

## 6. Conclusions and Future Directions

Advances in modern genetics have provided us with unprecedented opportunities to explore the molecular genetics involved in diverse neurodegenerative diseases. As the genomic revolution unfolds, novel genetic risk factors implicated in the pathogenesis of atypical parkinsonism syndromes emerge, providing us with crucial insights into intricate relationships among the complex spectrum of neurodegenerative diseases. In the near future, it is hoped that these molecular genetic signatures will guide clinicians in establishing an accurate clinical diagnosis, inform clinical decision-making, allow us to classify disease based on molecular deficits and highlight targets for rational therapeutic interventions.

In the near future, the major focus of genomic research in atypical parkinsonism is to further dissect pathways implicated in the pathophysiology, particularly those shared by several neurodegenerative diseases. Our current, although limited, understanding of the genetics of atypical parkinsonism already highlights common overlapping themes, such as a propensity to form protein aggregates, impairment in intracellular trafficking and dysfunctional protein degradation. These commonalities support the notion that atypical parkinsonism syndromes are part of a disease spectrum, and it is hoped that therapeutic strategies targeted against these shared pathways could be applicable to a wider range of neurodegenerative diseases.
